# Applying machine learning to optical coherence tomography images for automated tissue classification in brain metastases

**DOI:** 10.1007/s11548-021-02412-2

**Published:** 2021-05-30

**Authors:** Jens Möller, Alexander Bartsch, Marcel Lenz, Iris Tischoff, Robin Krug, Hubert Welp, Martin R. Hofmann, Kirsten Schmieder, Dorothea Miller

**Affiliations:** 1 Photonics and Terahertz Technology, Ruhr University Bochum, Bochum, Germany; 2 Department of Neurosurgery, University Hospital Knappschaftskrankenhaus Bochum, Ruhr University Bochum, Bochum, Germany; 3 Department of Pathology, University Hospital Bergmannsheil Bochum, Ruhr University Bochum, Bochum, Germany; 4 Technische Hochschule Georg Agricola, Bochum, Germany

**Keywords:** Metastases, Optical coherence tomography, Automated tissue differentiation, Histopathology, Computational diagnostics, Machine learning

## Abstract

**Purpose:**

A precise resection of the entire tumor tissue during surgery for brain metastases is essential to reduce local recurrence. Conventional intraoperative imaging techniques all have limitations in detecting tumor remnants. Therefore, there is a need for innovative new imaging methods such as optical coherence tomography (OCT). The purpose of this study is to discriminate brain metastases from healthy brain tissue in an ex vivo setting by applying texture analysis and machine learning algorithms for tissue classification to OCT images.

**Methods:**

Tumor and healthy tissue samples were collected during resection of brain metastases. Samples were imaged using OCT. Texture features were extracted from B-scans. Then, a machine learning algorithm using principal component analysis (PCA) and support vector machines (SVM) was applied to the OCT scans for classification. As a gold standard, an experienced pathologist examined the tissue samples histologically and determined the percentage of vital tumor, necrosis and healthy tissue of each sample. A total of 14.336 B-scans from 14 tissue samples were included in the classification analysis.

**Results:**

We were able to discriminate vital tumor from healthy brain tissue with an accuracy of 95.75%. By comparing necrotic tissue and healthy tissue, a classification accuracy of 99.10% was obtained. A generalized classification between brain metastases (vital tumor and necrosis) and healthy tissue was achieved with an accuracy of 96.83%.

**Conclusions:**

An automated classification of brain metastases and healthy brain tissue is feasible using OCT imaging, extracted texture features and machine learning with PCA and SVM. The established approach can prospectively provide the surgeon with additional information about the tissue, thus optimizing the extent of tumor resection and minimizing the risk of local recurrences.

## Introduction

Metastases are the most common intracerebral tumors. They are increasingly observed in recent years, which may be caused by an aging population or easier detection due to improved diagnostic imaging [1, 2]. Even though the prognosis remains poor, microsurgical resection is related to a prolonged survival of the patient [3]. Despite advances in microsurgical techniques, the risk of local recurrence after surgery without adjuvant therapy approaches 50–60% at one year [4]. Adjuvant focal radiotherapy or whole brain radiation significantly increase local control but may be associated with treatment related toxicity [4, 5].

Remaining contrast enhancement on early postoperative MRI can be detected after metastasectomy in up to 20% of cases and is significantly associated with local recurrence [6]. Some authors therefore suggest supramarginal resection or en-bloc resections with tumor-free margins [6–8]. However, this is not always feasible depending on the eloquence of the location or the size of the tumor. Neurological impairment can occur and negatively impact quality of life [9, 10]. Thus, exact intraoperative differentiation between tumor and healthy brain tissue is essential.

Intraoperative imaging, such as intraoperative ultrasound (iUS) or intraoperative MRI (iMRI) are able to detect macroscopic tumor remnants with good sensitivity and specificity but are unable to discriminate tumor from healthy tissue on a submillimeter-level [11–13]. Also, the acquisition of MRI images is time-consuming and increases surgery time [14]. A fast imaging modality that can detect minimal tumor remnants could improve surgical outcome. For this purpose, innovative imaging techniques, such as optical coherence tomography (OCT) have been developed [15, 16].

OCT is already an established method in ophthalmology and dermatology for the microscopic detection of pathological tissue and is used in medical research in dermatology [17, 18], gastroenterology [19–21] and urology [22].

Several groups have shown, that OCT can distinguish between tumorous and healthy brain tissue in an ex vivo as well as intraoperative setting [23–25]. The evaluation of OCT images, however, remained somewhat subjective in these studies. Only very few studies have used a quantitative approach mainly based on the optical attenuation [26]. However, the attenuation coefficient of tissue changes with the wavelength and thus depends on the spectral distribution of the specific light source in the system.

Lenz et al. suggested an alternative texture-based automated classification algorithm that is independent of the light source relying on structural features in the acquired and processed OCT images. The classification algorithm could distinguish healthy brain tissue from meningioma with an accuracy of 98%, showing that automated classification of tissue using OCT in a clinical environment is feasible [27]. Meningioma tissue was chosen for this initial analysis for its imaging properties being easily distinguishable from brain tissue. However, this might not be of clinical importance.

Analyzing the morphological characteristics of metastases visualized by OCT seemed a logical consequence and of greater clinical interest. The primary objective of this study is to show that OCT is able to acquire imaging information of metastases enabling the automated discrimination of necrotic tissue and vital tumor against healthy brain tissue. Furthermore, we wanted to show that the application of various image processing techniques including automated segmentation of OCT images, determination of texture parameters and machine learning can lead to automated tissue classification. Finally, the aim is to provide reliable evidence of the potential role of OCT in intraoperative imaging and automated discrimination of various tissue types in metastases.

## Methods

### Tissue resection

The study was approved by the local ethics committee. Patients gave informed consent to the study prior to surgery.

Tissue samples of approximately 5 mm in diameter and 2–3 mm in thickness were taken from the tumor during surgery. In case of a subcortical location of the metastasis, a sample of healthy brain tissue was taken from the access path. Fresh samples covered in normal saline were directly given to OCT scanning (see below) and examined by an experienced neuropathologist thereafter. All tumor tissue not used for the study was directly subjected to pathological examination.

The study collective consisted of 22 tissue samples from 20 patients. Patient histology included fifteen pulmonary carcinomas (seven adenocarcinomas, four squamous cell carcinomas and four small cell lung cancers), three adenocarcinomas of the breast, one colorectal adenocarcinoma and one laryngeal squamous cell carcinoma.

### OCT

A commercially available spectral domain high-resolution OCT system (Thorlabs Ganymede, GAN210C1) was used for OCT imaging. Based on a broadband light source in the near-infrared range (central wavelength: 930 nm) the system provides an axial resolution of 6 µm and a lateral resolution of 8 µm. Maximal penetration depth of the system is 2.7 mm in air and approximately 2 mm in brain tissue, depending on the tissue characteristics, especially the refractive index of human brain tissue which is approximately 1.36 [28].

One acquisition of the OCT System can obtain one entire depth scan of the sample (A-scan) while the light beam penetrates the sample at one spot of the surface. By scanning along a line across the surface of the sample, a two-dimensional image consisting of several A-scans can be acquired (B-scan). Finally, utilizing additional scanning in a direction perpendicular to the previous enables acquisition of volumetric OCT images. All samples were imaged with a resolution of 1024 × 1024 × 1024 pixels scanning a surface of 3.5 × 3.5 mm^2^ and an imaging depth of 2 mm. As a result, more than 1000 B-scans with a resolution of approximately one megapixel were acquired for each sample. The acquisition time for a full volumetric image is approximately 36 s (A-scan rate: 29 kHz).

## Histopathology

After imaging the samples with the OCT system, samples were fixed in formalin (4.0%) and embedded in paraffin. Slices of 3 µm thickness were processed and stained with hematoxylin and eosin. As a reference for the automated tissue classification, the slices were then histologically evaluated by an experienced neuropathologist in order to determine tumor entity. Furthermore, the percentage of vital tumor, necrosis and healthy brain tissue was established manually for each sample.

Only those tissue samples were included in the further analysis that showed at least 60% of either vital tumor or necrotic tissue or at least 90% healthy tissue on histology. Thus, 10 tumor samples and 4 samples consisting of healthy tissue were used for tissue discrimination (see Table 1).

**Table 1:** Study collective showing the percentage of vital tumor, necrosis and healthy tissue as determined by histopathologic findings for all samples that were taken into account for the automated classification approach; italic numbers indicate the main percentage of tissue type present which was used as label for the classification with support vector machines (SVM)

Label for classification	Patient diagnosis		Histology of sample		
	Primary tumor	Tumor histology	Tissue percentage		
			Vital tumor	Necrosis	Healthy tissue
Necrosis	Lung	Adenocarcinoma	0	*100*	0
	Lung	Squamous epithelial tumor	0	*100*	0
	Lung	Squamous epithelial tumor	0	*100*	0
	Lung	Squamous epithelial tumor	0	*100*	0
	Colon	Adenocarcinoma	0	*100*	0
	Breast	Adenocarcinoma	0	*100*	0
Vital tumor	Lung	SCLC	*70*	30	0
	Lung	Adenocarcinoma	*70*	0	30
	Lung	Adenocarcinoma	*60*	5	35
	Breast	Adenocarcinoma	*95*	5	0
Healthy tissue	Lung	Adenocarcinoma	0	0	*100*
	Lung	Squamous epithelial tumor	0	0	*100*
	Lung	SCLC	10	0	*90*
	Breast	Adenocarcinoma	0	0	*100*

*SCLC* small cell lung cancer

Resected tumor tissue that was not used for the OCT-study was examined using standard histopathological and immunohistochemical staining. Further molecular analysis was performed, if required clinically.

## Texture analysis and tissue classification

**Fig. 1:**
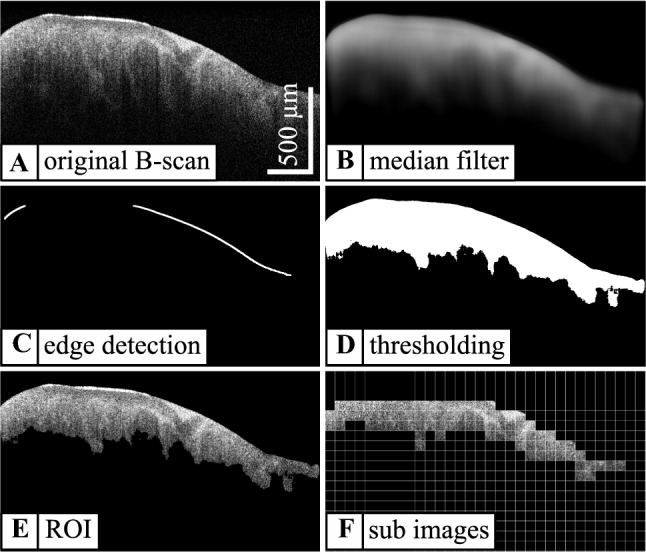
Exemplary visualization of the automated segmentation shown for a B-scan of tumorous tissue (necrosis) comprising **a** the originally acquired B-scan **b** median filtering **c** edge detection **d** thresholding **e** determination of the region of interest **f** resulting sub images used for texture feature extraction

**Fig. 2:**
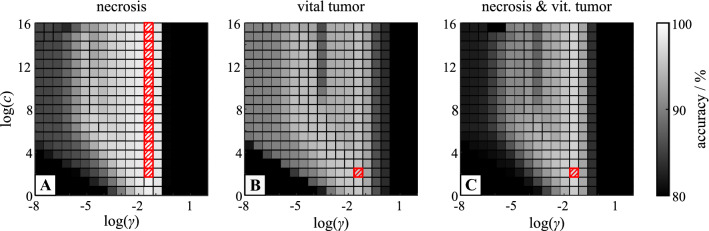
Results of the grid searches for the best SVM classification accuracy achieved for the three studied tissue types **a** necrosis **b** vital tumor **c** combination of necrosis and vital tumor; resulting from the classification using all available texture parameters (LBP + RL + H + L) and z-score normalization; parts of the grid search achieving the absolute maximum accuracy (see Table 2) are hatched

Image segmentation, texture analysis, principle component analysis (PCA) and support vector machine (SVM) were applied as previously described [27]: First, a segmentation algorithm was used which extracts relevant sub images from each B-scan. Figure 1 illustrates the processing steps from the original B-scan to the segmented image, including median filtering to enable a smooth edge detection, followed by Canny edge detection [29], thresholding using Otsu’s method to determine the region of interest (ROI) [30]. Even in cases when the edge detection is not continuous (see Fig. 1) or multiple edges are detected, the combination of edge detection and thresholding provides reasonable results. The original B-scan was then sub-divided into 32 × 32 pixel sub-images. The uppermost three rows of sub-images were removed to exclude image artifacts originating from the DC part in the iFFT for image generation. All remaining sub-images lying at least 90% within the ROI were used as a basis for the determination of all texture parameters used in this study. Additionally, influences of system parameters like the confocal point spread function were evaluated exemplary, by correcting all B-Scans prior to the texture analysis [31]. Corrections may improve the robustness of our classification approach but do not change the best classification accuracy achieved by the introduced method. The influence of imaging artifacts like specular reflections was found to play a minor role in the texture analysis. All segmented B-Scans were inspected visually beforehand and an estimated percentage of less than 0.1% of the used sub images contained artifacts. Taking the averaging of each texture feature along one B-Scan into account, artifacts do not noticeably impact the classification results. To quantify the structural information in the acquired OCT images, four different texture parameters were used: local binary patterns (LBP; 256 features) [32], run length analysis (RL; 7 features) [33], Haralick's texture features (H; 5 features) [34] and Laws' texture energy measures (L; 8 features) [35]. These parameters represent a great variety of structural and statistical features including local gray value distribution, local brightness, directional structural changes and more complex structural features. Given the heterogeneous structure of tumor tissue, covering different types of structural features in the analysis seemed target-oriented and already led to high classification accuracies with meningioma. Each feature was averaged over one entire B-scan while every acquired three-dimensional OCT image contains 1024 B-scans each. All computed data sets were normalized by a z-score normalization which makes the data set mean-free and scales it to a standard deviation of one [36]. A principal component analysis (PCA) was then applied to the data set with z-score normalization as well as to the data set without z-score normalization reducing the dimensionality and thus also the size of the resulting data sets. As a final step, a support vector machine (SVM) was utilized to discriminate tumorous tissue (see Table 2) from healthy tissue [37, 38]. To achieve reliable results, a grid search was implemented which performs the classification for a certain range of the cost parameter c and the kernel width $\gamma$. The cost parameter c was varied between 1 and ${10}^{16}$, whereas the kernel width $\gamma$ was varied between ${10}^{-8}$ and ${10}^{2}$ (see Fig. 2). To make the best possible statistical use of the small available data set, a tenfold cross validation was performed varying training and test data sets for each of the ten classification runs. Textural features of all B-scans are allocated to either the training data set or the test data set. Preventing redundant features in training and test data set provides an unambiguous classification model. Thus, a real and unbiased classification accuracy can be obtained.

**Table 2:** The table shows the obtained classification accuracies (in percent) depending on the used set of texture features, application of a *z*-score normalization and the tissue type that should be discriminated from healthy tissue

Tissue type (no. samples)	Vital tumor (4)		Necrosis (6)		Vital tumor and necrosis (10)		Averaged	
Used texture features	w/o *z*-score	w/ *z*-score	w/o *z*-score	w/ *z*-score	w/o *z*-score	w/ *z*-score	w/o *z*-score	w/ *z*-score
L	93.05	92.25	94.73	92.68	93.02	92.13	93.6	92.35
H	88.47	88.02	88.84	89.03	88.71	88.96	88.67	88.67
H + L	93.06	89.82	94.74	88.52	93.04	88.88	93.61	89.07
RL	88.89	88.75	90.5	90.58	90.11	90.37	89.83	89.9
RL + L	91.79	93.04	93.29	87.87	91.73	89.52	92.27	90.14
RL + H	88.93	87.88	90.56	87.47	90.08	88	89.86	87.78
RL + H + L	91.79	92.7	93.27	87.72	91.76	88.58	92.27	89.67
LBP	92.65	93.68	97.8	98.9	94.19	95.14	94.88	95.91
LBP + L	93.18	**95.3**	94.93	**99.08**	93.12	**96.56**	93.74	**96.98**
LBP + H	92.66	94.27	97.81	98.86	94.17	95.68	94.88	96.27
LBP + H + L	93.18	**95.68**	94.93	99.01	93.12	**96.68**	93.74	**97.12**
LBP + RL	88.6	93.64	93.94	99.03	90.71	95.12	91.08	95.93
LBP + RL + L	91.92	**95.52**	93.69	**99.07**	92	**96.53**	92.54	**97.04**
LBP + RL + H	88.66	94.33	93.89	**99.06**	90.76	95.74	91.1	96.38
*LBP* + *RL* + *H* + *L*	91.92	***95.75***	93.69	***99.1***	92	***96.83***	92.54	***97.23***

The two rows on the right side represent the averaged classification accuracy that could be achieved for the given set of used texture features with and without z-score normalization. The four best classification accuracies per tissue type are marked bold and the texture parameter set with the best overall classification accuracy is marked bolditalic

## Results

**Fig. 3:**
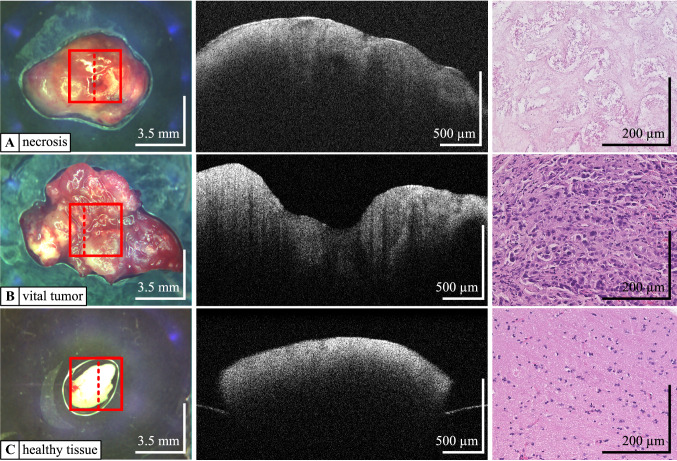
Examples of the analyzed tissue types **a** necrosis **b** vital tumor (metastasis of breast cancer) **c** healthy tissue. Left column photograph of the tissue sample. The dashed line in the photograph indicates the real position of the displayed OCT B-scan; the square marks the surface of the entire volumetric image that was acquired Middle column OCT B-scan; please note the smooth and regular appearance of the healthy tissue scan (**c**) as compared to the irregular appearance of necrosis (**a**) and vital tumor (**b**) Right column corresponding histology slide (hematoxylin–eosin staining, 200 × magnification); please note the acellularity and empty spaces of the necrotic tissue (**a**) and the high cellularity of the tumor tissue (**b**) as compared to the structured and regular appearance of cortical tissue featuring mainly glial cells (**c**)

The unprocessed OCT images as seen in Fig. 3 already show differences in the morphology of the individual tissue samples, but they do not allow an exact tissue discrimination by the surgeon. Validated by histological examination, the automated tissue classification allows a precise classification of vital tumor, necrosis and healthy brain tissue. By combining all texture parameters used, we achieved a classification accuracy between vital tumor and healthy tissue of 95.75%. Furthermore, we were able to distinguish healthy tissue from necrosis with an accuracy of 99.10%.

A generalized discrimination between tumorous tissues (vital tumor and necrosis) and healthy tissue led to a classification accuracy of up to 96.83%. It is noticeable that the combination of LBP, RL, H and L (with z-score normalization) allows the most accurate differentiation for all tissue discriminations performed. Nonetheless, classifications using a fraction of all texture parameters achieve equally significant results as presented in Table 2. Consideration of fewer parameters leads to less data that has to be analyzed, resulting in shorter computation times (see Table 3) and prospectively improved intraoperative applicability.

**Table 3:** The table shows measured computation times comprising average time per B-scan and total time for a 3D image for each step of the texture analysis. Additionally, the average times for the PCA and prediction of a class for a 3D image/sample using SVM are shown. Compared to the processing time required for the texture analysis, times of PCA and SVM are negligible

Processing step	Average time/B-scan	Total time/3D image
Segmentation	597 ms	591.8 s
Haralick features	142 ms	141.1 s
LBP features	41 ms	40.2 s
Laws features	160 ms	158.5 s
Run length features	666 ms	660.4 s
Total texture analysis	1606 ms	1592.0 s
PCA	–	0.032 s
SVM	–	< 1.2 s

The best values for classification accuracy in Table 2 were determined by a grid search performed for each set of texture parameters. The corresponding results of the grid search depending on cost parameter c and kernel width $\gamma$ are depicted in Fig. 2. Evaluation of the grid search results can be executed in different ways: the spot with maximum accuracy or the area of the grid that provides an accuracy higher than a threshold (in this case set to 90%). Ultimately, both approaches lead to the same result. Combining LBP, RL, H and L and *z*-score normalization results in maximum accuracy and widest range of c and $\gamma ,$ leading to an accuracy of more than 90%.

## Discussion

In our study, we investigated a method of automated classification of brain metastases and healthy brain tissue using optical coherence tomography. OCT is a promising new intraoperative imaging technique in neurosurgery that has the potential to improve resection and thus survival in patients with brain metastases. The achievable resolution of OCT in the micrometer range might outperform alternative techniques, such as iMRI, iUS and fluorescence guidance.

Our study is related to the work of various other groups that focus on distinguishing brain metastases or glioma from brain tissue by using OCT: while most groups rely on visual inspection in comparison to histology [39, 40], other groups use optical parameters such as the optical attenuation coefficient [26, 41, 42], birefringent properties [43] or information provided by speckle in OCT images [44, 45] to identify differences between tissue types to be distinguished. Alternative approaches include the application of different classifiers for tissue analysis based on previously extracted morphological features from OCT images [46]. Ultimately, Gossage et al. analyzed structure and speckle in OCT images and used a Bayesian classification model to prove general feasibility of tissue classification using texture analysis [47, 48].

In comparison to previously named approaches using the optical attenuation coefficient to distinguish between different tissue types, our approach is based on an automated classification using a rather sophisticated texture analysis. The optical attenuation coefficient is one single tissue parameter whose physical origin is well understood. Therefore, tissue types that have different attenuation properties can simply be classified by determining a threshold for the attenuation coefficient. For tissue types that do not differ in optical attenuation, these approaches fail. The advantage of our approach is the broad range of texture parameters that are taken into account by utilizing a variety of structural and statistical features. Using this approach, different tissue characteristics that result in different image patterns can potentially be used to differentiate tissue types, without analyzing the physical effects behind them. Instead of a single optical parameter like the optical attenuation coefficient, a great number of texture features is extracted from the OCT images. Because of the high number of extracted features, determining a simple threshold for classification is no further applicable. As a result, reduction of the dataset by PCA and classification using SVM was used to handle the increased amount of data and to ultimately enable precise discrimination between tumorous and healthy tissue. Lenz et al. first tested the approach on meningioma tissue [27]. The aim of the current study was to validate this approach in a clinically more relevant setting, that is differentiating brain metastases from healthy brain tissue.

Thus, our approach shows that classification of different tissue types ex vivo is possible. All methods used could be translated to a fast, intraoperative approach, which could provide additional information about the present tissue to support the surgeon's decision during resection.

Various developments demonstrate that the intraoperative implementation of OCT is feasible [49]. Innovative approaches enable contact-free imaging of the tissue and can thus minimize the risk of intraoperative infections. Also, real-time acquisition of OCT images enables the surgeon to collect data concerning the tissue microarchitecture immediately before, during and after tissue manipulation.

By combining an intraoperative OCT and the classification algorithm presented by us, an entire resection cavity could be imaged, segmented and classified.

Occasionally, pathologists examine tissue samples intraoperatively to evaluate resection margins or during stereotactic biopsies. This procedure ties up staff, is time-consuming and might result in an extended surgery time.

To put a potential application of our classification approach into perspective, it is conceivable that the surgeon provides a tissue sample of interest for OCT examination during surgery. This sample could then be rapidly segmented and classified in order to give the surgeon helpful information about the entity of the imaged and analyzed tissue. Due to the analysis method based on B-scans, the evaluation and classification of the imaged tissue sample is independent of the OCT system used and thus highly flexible. Nevertheless, further investigations are needed to confirm the independence of different acquisition parameters as for example wavelength and resolution of the used imaging system.

**Fig. 4:**
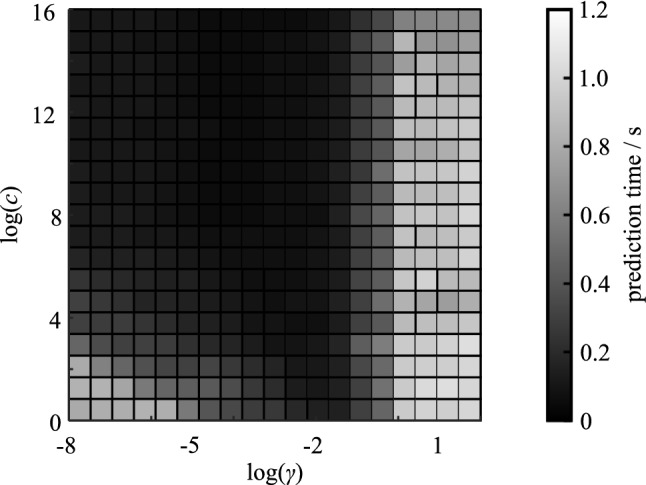
Prediction times of the SVM to classify a 3D data set with respect to the previously calculated classification model; the prediction does not exceed 1.2 s regardless of the combination of parameters c and $\gamma$; especially areas of the grid search that provide accurate classification (see Fig. 2) result in rapid classification

The potential application of our classification approach in an intraoperative scenario introduces some additional requirements to the used algorithm. Especially computation time plays a big role, when trying to maintain the initial surgery time. The introduced classification method can be divided into three major steps of computation: calculation of texture parameters, creating the classification model and finally the classification of unknown tissue into one of the two classes of the model. The computation of the classification model requires consideration of the whole study collective in order to obtain the best classification accuracy possible, which leads to high computation times. This process can be executed prior to the intraoperative use of the approach. Therefore, classification of a single OCT 3D image just consists of the calculation of texture parameters and then applying the already known classification model to the acquired data set. In this manner, the processing time for a single sample can drastically be reduced. Table 3 gives an overview of all computation times resolved for each step of the analysis and classification. Given the processing times of 32 ms for the PCA and less than 1.2 s for the SVM, the extraction of all texture features from the 3D OCT image is clearly the limiting factor for a real time application. The algorithm implemented for texture analysis is rather focused on being a proof of concept than achieving high performance. Accordingly, the process can potentially be accelerated by multiple factors. Figure 4 exemplary shows the prediction times of the SVM for the classification of one 3D image. Especially areas of the grid search that lead to high classification accuracies (see Fig. 2) provide rapid classification times of the SVM.

We are aware that our sample collective is relatively small. However, the study collective represents some of the most common metastatic tumor types seen in our neuro-oncology service, including lung, breast and colorectal carcinoma. Moreover, each data set consists of more than 1000 B-Scans. We applied a tenfold cross validation, which varies training and test data sets to determine an improved classification model. By combining ten independent classification models, we aimed to improve the quality of the results for the available sample collective. Even though the tissue samples originate from metastases of various primary tumors, a classification against healthy tissue was possible. This suggests that all imaged metastases have optical properties that deviate from the optical properties of healthy brain tissue and can therefore be distinguished from the latter using our classification approach. Nevertheless, our machine learning approach will need additional validation on a larger and more varied number of metastases in the future.

OCT is an optical imaging technique that does not require tissue contact and can be applied intraoperatively. Competing noninvasive imaging techniques can be divided into established intraoperative approaches, such as sonography and MRI, and more experimental approaches, like Raman spectroscopy. While tissue contact is mandatory for sonography, MRI is accompanied by long acquisition times and a sophisticated setup. On the other hand, Raman spectroscopy is at the same stage as OCT but brings additional restrictions to the surgery because of its sensitivity to external influences like ambient light. Conceptually, OCT can provide 3D imaging of tissue without the need for any changes to the resection cavity and its surroundings.

Support Vector Machines (SVM) were originally designed to enable classification by dividing a data set into two subsets. In this study it is shown that a classification between tumorous and healthy tissue is possible which can provide additional, precious information for the surgeon during tumor resection (one vs. rest approach). Furthermore, discrimination of necrosis from healthy tissue as well as vital tumor from healthy tissue is achieved with high accuracy (one vs. one approach). Nevertheless, determining the percentage of more than two tissue types (i.e. vital tumor, necrosis and healthy tissue) is desirable. While the presented approach can be used as evidence that different tissues can be distinguished, analysis could be processed for smaller sub volumes of the sample in the future and thus, provide classification of smaller volumes resulting in higher resolution and potentially a determination of tissue type percentages.

Consequently, combining OCT as imaging technique with the presented tissue classification approach, leads to a promising tool that potentially can support brain tumor surgeries in the future. Thus, tumor remnants could be reduced while at the same time protecting the surrounding healthy tissue by highly resolved classification of tissue using the proposed methods.

## Conclusions

Complete tumor resection, the recognition of tumor boundaries and the associated protection of healthy brain tissue remains a major challenge in neurosurgery. This study convincingly demonstrates that the application of texture analysis on OCT images enables a precise classification of tumorous and healthy tissue in brain metastases. The establishment of this procedure can prospectively provide the surgeon with additional information about the tissue, optimizing the extent of tumor resection and thus minimize the risk of local recurrences.

## Data Availability

The datasets generated during and/or analyzed during the current study are available from the corresponding author on reasonable request.

## Declarations

### Code availability

Code for data cleaning and analysis are available from the corresponding author on reasonable request.
